# A Couple-Based Intervention for Chinese Older Adults With Type 2 Diabetes: A Randomized Clinical Trial

**DOI:** 10.1001/jamanetworkopen.2024.52168

**Published:** 2025-01-02

**Authors:** Conghui Yang, Jingyi Zhi, Yingxin Xu, Xinyu Fan, Xueji Wu, Dong Roman Xu, Jing Liao

**Affiliations:** 1 Department of Medical Statistics and Epidemiology, School of Public Health, Sun Yat-sen University, Guangzhou, China; 2 Sun Yat-sen Global Health Institute, School of Public Health and Institute of State Governance, Sun Yat-sen University, Guangzhou, China; 3 Department of Integrated Profession Management, Guangzhou Center for Disease Control and Prevention, Guangzhou, China; 4 Acacia Lab for Implementation Research, Southern Medical University Institute for Global Health and Center for World Health Organization Studies, School of Health Management and Dermatology Hospital of Southern Medical University, Guangzhou, China

## Abstract

**Importance:**

Spousal involvement in diabetes care is recommended theoretically, but effectiveness in clinical settings and among diverse populations is unclear.

**Objective:**

To test the effect of a couple-based intervention among Chinese older patients with type 2 diabetes and their spouses.

**Design, Setting, and Participants:**

This multicenter randomized clinical trial comprised 2 arms: a couple-based intervention arm and an individual-based control. The trial was conducted across 14 community health care centers in Guangzhou, China, between September 1, 2020, and June 30, 2022, and included patients with confirmed type 2 diabetes aged 55 years or older. Eligible partners were married to or cohabitated with the patients. Patients who previously participated in type 2 diabetes education courses were excluded, as were couples who both had diabetes, to make a clear distinction between patients and spouses. The data were analyzed between January 2023 and April 2024.

**Interventions:**

The interventions consisted of 4 weekly group education sessions followed by behavior change booster telephone calls over 2 months that targeted either patients and spouses (ie, intervention arm) or patients alone (ie, control arm). Follow-up assessments were conducted at 6 and 12 months post intervention.

**Main Outcomes and Measures:**

The primary outcome was hemoglobin A_1c_ (HbA_1c_) levels for patients and quality of life for their spouses. Collective efficacy and behaviors were secondary outcomes for both patients and spouses. Group comparisons were conducted using multilevel models based on an intention-to-treat approach, with outcome measures assessed for both patients and their spouses.

**Results:**

A total of 207 couples were included in the study, with 106 randomized to the intervention arm and 101 to the control arm. The mean (SD) age of patients was 66.0 (6.5) years, with 105 (50.7%) being men; spouses had similar demographics. Patients’ HbA_1c_ levels decreased in both arms over the 12-month follow-up, with no significant between-arm differences (β = −0.08; 95% CI, −0.57 to 0.42). Collective efficacy and collective behavior for patients increased after intervention but with a similar magnitude between arms. None of these measures showed between-arm differences among spouses in either arm. In subgroup analysis, decreases in HbA_1c_ levels were constant and lasting in patients with high baseline HbA_1c_ levels (≥8.0%) with a statistically significant difference.

**Conclusions and Relevance:**

These findings show that the overall treatment effect of the couple-based intervention was weak. However, the couple-based intervention benefited patients with poor glucose control. Patients’ glucose levels, spouses’ availability to provide support, and couples’ collaborative preferences for mutual or individual diabetes management should be considered in tailoring treatment strategies among older adults with type 2 diabetes.

**Trial Registration:**

Chinese Clinical Trial Registry Identifier: ChiCTR1900027137

## Key Points

**Question:**

Is a couple-based intervention more effective than an individual-based intervention for managing type 2 diabetes in Chinese older adults?

**Findings:**

In this randomized clinical trial of 207 pairs of adults with type 2 diabetes and their spouses assigned 1:1 to a couple-based intervention arm or an individual-based control arm, patients’ hemoglobin A_1c_ (HbA_1c_) decreased in both arms with no significant between-arm differences. However, more evident decreases in HbA_1c_ were shown among patients with high baseline HbA_1c_ and lasted for 12 months.

**Meaning:**

These findings suggest that a couple-based intervention may be more beneficial than an individual-based intervention for older adults with poorly controlled HbA_1c_.

## Teaser

This randomized clinical trial tests the effect of a couple-based intervention among older Chinese patients with type 2 diabetes and their spouses.

## Introduction

China has the largest number of patients with diabetes, reaching nearly 141 million cases in 2021, of which type 2 diabetes accounts for more than 90%.^1^ Although the Chinese government aims to control and treat diabetes primarily by community-based health care services, the awareness, treatment, and control rates of diabetes are still low at less than 37%, 33%, and 50%, respectively.^2^ The reasons for these low rates may lie in the shortage of skilled community health care workers^3^ and poor self-management generally associated with poor knowledge among older adults with type 2 diabetes.^4^ Managing type 2 diabetes is complex and requires constant monitoring. Given that daily management of diabetes occurs within the family, recent studies^5^ and clinical guidelines^6^ have increasingly emphasized the key role of family members, especially spouses.^7^

Although spousal involvement in diabetes care is recommended in the behavioral theory literature,^8,9^ the evidence derived from randomized clinical trials (RCTs) is unclear. Few RCTs have included an individual-based intervention comparator, using instead a blank control condition, making it impossible to draw a conclusion on the net effect of couple-collaborative vs individual-based management of diabetes care.^10^ Moreover, most of these studies reported behavioral and psychological outcomes, while evidence on objective clinical outcomes is lacking. Our review of the literature indicates that only 2 RCTs included blood glucose measures in examining couple-based interventions, both conducted in a US middle-aged population.^5,11,12^ Evidence on outcomes among spouses is limited, as most studies provided only patient outcomes.^5^ It is also unclear whether the effect of a couple-based intervention would vary by participants’ characteristics. Sex-specific findings have been shown previously,^13^ indicating that female patients may benefit more from a couple-based intervention than their male counterparts.^14^ Furthermore, patients with higher initial hemoglobin A_1c_ (HbA_1c_) levels may experience more improvement during the intervention.^11^ As current RCTs are mainly conducted among European and American older populations, the extent to which their findings apply to Chinese older couples remains to be studied.

Therefore, this study evaluated the effects of a multicentered couple-based intervention in promoting health and well-being of Chinese older adults (aged ≥55 years) with type 2 diabetes and their spouses. Our intervention was designed in accordance with the Couple-Based Collaborative Management Model (eFigure in Supplement 2), which has been applied to the management of several chronic diseases, including diabetes.^15^ The Couple-Based Collaborative Management Model is constructed on 2 main theories: the dyadic model of coping with chronic illness^16^ and social cognitive theory,^17^ positing that when couples view chronic illness as a shared problem requiring joint coping, their collective efficacy—defined as their mutual belief in the ability to cooperate in disease management—will be enhanced, resulting in collective behavior changes. Consequently, we hypothesized that the intervention would strengthen collective efficacy and trigger collective behavior changes and ultimately improve the patient’s clinical outcomes and the couple’s quality of life.

## Methods

### Trial Design and Procedure

This multicenter RCT was conducted across 14 community health care centers in Guangzhou, China, between September 1, 2020, and June 30, 2022. The study protocol was published previously^7^ and is included in Supplement 1. The study was approved by Sun Yat-sen University’s institutional review board, and written informed consent was obtained from all participants. All study procedures were conducted in accordance with the guidelines of the Declaration of Helsinki.^18^ We report the study findings in accordance with the Consolidated Standards of Reporting Trials (CONSORT) reporting guideline for RCTs.

Briefly, older adults with type 2 diabetes and their spouses eligible for the study were included and randomly assigned 1:1 to the couple-based intervention arm or the individual-based control arm within each center after the baseline assessment. The stratified randomization by patient sex and age was conducted by a statistician not involved in the project.

Interventions consisted of weekly group education and training sessions delivered at the health care centers in the first month, followed by 2 months of weekly, tailored behavior change booster calls that targeted couples (ie, intervention arm) or individual patients (ie, control arm). Face-to-face follow-up assessments were conducted by research assistants (C.Y., J.Z., and Y.X.) at 6 and 12 months after baseline.

### Participants

Patients included in the study were adults who (1) had confirmed type 2 diabetes and were registered for National Essential Public Health Services for type 2 diabetes management; (2) had a fasting blood glucose level greater than 8.0 mmol/L, an HbA_1c_ level greater than 7.0% (53 mmol/mol), or newly diagnosed type 2 diabetes during the past 12 months; (3) were aged 55 years or older; (4) had basic literacy and adequate cognitive and physical capability; (5) were living with a spouse; and (6) were willing to provide informed consent to participate in the study. Patients who previously participated in type 2 diabetes education courses were excluded. We set the age cutoff at 55 years to increase our targeted population by including those who are less likely to be widowed and easier to recruit as indicated by our group’s pilot study.^19^

Eligible spouses were (1) married to or cohabitated with an adult with type 2 diabetes, (2) without mental or physical dysfunction that may interfere with the study, and (3) willing to provide informed consent. We excluded couples who both had diabetes for a clear distinction between patients and spouses.

### Interventions

The intervention and control arms involved 4 weekly group education and training sessions and received behavior change booster calls over the following 2 months. The educational content was mainly based on the type 2 diabetes management program,^20^ covering topics of diabetes and complications, healthy diet, medication, exercise, and addressing behavior change techniques.^21^

#### Group Education and Training

The 4 weekly 2-hour group education and training sessions included 8 to 10 patients or couples and were facilitated by 2 care managers. At the end of each session, participants set health-related behavior goals and developed plans for the following week with collaborative implementation intentions,^22^ which would be reviewed by the group in the next session.

For the intervention arm, patients and spouses were encouraged to participate in the sessions together. The education and training components were framed based on the couple’s issues whenever possible, and couple-level discussions and practices were interspersed throughout the sessions. The couples were encouraged to share their pragmatic strategies for coping with type 2 diabetes. The care managers provided spouses with knowledge and techniques on how they could help the patient manage their diabetes.

For the control arm, only the patients participated in the group education and training and set personal goals. Spouses, other family members, or friends did not attend these sessions.

#### Behavior Change Booster Calls

Personalized telephone calls were conducted to address participants’ behavior change barriers, and call frequency varied by patients’ progress. In the first session, researchers asked whether patients had completed the behavioral changes associated with the 4 education and training sessions. If so, they were encouraged to continue to adhere to the program. If not, the researchers provided personalized guidance based on patients’ present dietary habits and remind them to control the diet according to the established plan. Follow-up calls were based on participants’ performance and targeted behaviors that needed further improvement. If patients fulfilled all the behavior change targets, researchers assessed only their maintenance status after another 3 weeks. In the intervention arm, both patients and their spouses were contacted at the same time and were encouraged to review their progress jointly, while only the patients received the telephone booster for the control arm.

### Outcomes

#### Health Outcomes

The primary outcome for the patients was HbA_1c_ level, which indicates average blood glucose levels over the preceding 2 to 3 months^23^ and is the criterion standard for measuring blood glucose control. For spouses, the primary outcome was health-related quality of life as measured using the 36-Item Short Form Health Survey (SF-36).^24^ The SF-36 contains Physical Component Summary (PCS) and Mental Component Summary (MCS) measures. The PCS measure includes scales for physical functioning, physical role, bodily pain, and general health, while the MCS measure comprises vitality, social functioning, emotional role, and mental health. Both are scored on a scale of 0 to 100, with higher scores indicating better quality of life. This measure was considered as the secondary outcome for patients.

#### Collective Efficacy

Collective efficacy was measured for both patients and spouses as an extension of self-efficacy^7^ using the Chinese version of the Diabetes Management Self-Efficacy Scale (C-DMSES).^25^ The 20-item C-DMSES assesses patients’ confidence in performing daily diabetes management activities from 0 (cannot do at all) to 10 (certainly can do). Spouses were also assessed using a modified version of the C-DMSES to evaluate their efficacy in assisting patients in daily diabetes management on the same 11-point scale.^26^

#### Collective Behavior

Patients’ diabetes self-management behavior was examined using the Chinese version of the Summary of Diabetes Self-Care Activities (SDSCA) questionnaire.^27^ The SDSCA contains 12 items that examine the number of days patients performed self-care activities, with higher scores indicating more days fulfilling the requirement. Physical activity behavior for both the patients and their spouses was measured using the International Physical Activity Questionnaire–Short Form,^28^ which evaluates the frequency and duration of 4 types of physical acidities over the past week and can be converted to metabolic equivalent task minutes per week based on published equations.^29^

### Statistical Analysis

The data were analyzed between January 2023 and April 2024. As stated in our study protocol (Supplement 1),^7^ the study sample size calculation was based on the patients’ primary health outcome of HbA_1c_. To detect a clinically meaningful change in glycemic control, a 0.5% or higher reduction in HbA_1c_ in the couple-based intervention arm vs the individual-based control arm over 12 months would be assumed, resulting in a sample size of 194 with 80% power to detect a between-arm difference of this magnitude (SD, 1.5%). For a longitudinal design with 3 repeated measures, the within-person correlation between repeated measures was assumed at 0.5, with α set at .05 and a dropout rate of 10%. We eventually recruited 207 pairs of adults with type 2 diabetes and their spouses, which would allow us to detect between-arm difference of 0.48% in HbA_1c_ if other parameters remained the same.

The intention-to-treat approach was applied to examine the treatment effects. The multilevel model was used to compare within- and between-arm differences in all outcomes from baseline to months 6 and 12 separately for patients and spouses. Given the study’s block randomization design, we treated patients’ or spouses’ repetitive outcomes as level 1 with fixed effects only, and patients or spouses and their community health care centers as levels 2 and 3 with random effects. All models were adjusted for baseline demographic variables (ie, age, sex, education level, retirement status), as well as diabetes duration for patient outcomes with fixed effects only. The treatment effect was further tested in 2 preset subgroup analyses stratified by sex and patients’ baseline HbA_1c_ level (ie, <8.0% or ≥8.0% [64 mmol/mol]). Missing outcomes were multiply imputed under the missing at random assumption using chained equations.^30^ The imputation model was consistent with the model used for the outcome analyses. We used R, version 4.1.0 (R Foundation) for the analyses, with a 2-sided *P* < .05 considered significant.

## Results

### Participants’ Baseline Characteristics

A total of 207 couples were included in the study, with 106 randomized to the intervention arm and 101 to the control arm. Baseline characteristics are reported in Table 1. The mean (SD) age of the patients was 66.0 (6.5) years; 105 (50.7%) were men and 102 (49.3%) were women. Most of the participants were retired (182 [87.9%]), and the mean (SD) duration of diabetes was 8.3 (7.3) years. Spouses’ demographic characteristics were similar to the patients’. No clinically meaningful differences were found between arms among the baseline characteristics.

**Table 1.** Participant Baseline Characteristics by Treatment Arm

Characteristic	Mean (SD) or No. (%)					
	Patients			Spouses		
	Overall (n = 207)	Intervention arm (n = 106)	Control arm (n = 101)	Overall (n = 207)	Intervention arm (n = 106)	Control arm (n = 101)
Age, y	66.0 (6.5)	65.9 (6.8)	66.1 (6.1)	65.8 (7.3)	65.8 (7.3)	65.7 (7.4)
Sex						
Female	102 (49.3)	49 (46.2)	53 (52.5)	105 (50.7)	57 (53.8)	48 (47.5)
Male	105 (50.7)	57 (53.8)	48 (47.5)	102 (49.3)	49 (46.2)	53 (52.5)
Education						
Primary school or less	69 (33.3)	30 (28.3)	39 (38.6)	77 (37.2)	37 (34.9)	40 (39.6)
Secondary school	61 (29.5)	37 (34.9)	24 (23.8)	54 (26.1)	29 (27.4)	25 (24.8)
High school or higher	77 (37.2)	39 (36.8)	38 (37.6)	76 (36.7)	40 (37.7)	36 (35.6)
Diabetes duration, y	8.3 (7.3)	7.7 (6.8)	8.9 (7.8)	NA	NA	NA
Retired	182 (87.9)	94 (88.7)	88 (87.1)	169 (81.6)	86 (81.1)	83 (82.2)

Abbreviation: NA, not applicable.

**Figure 1.**
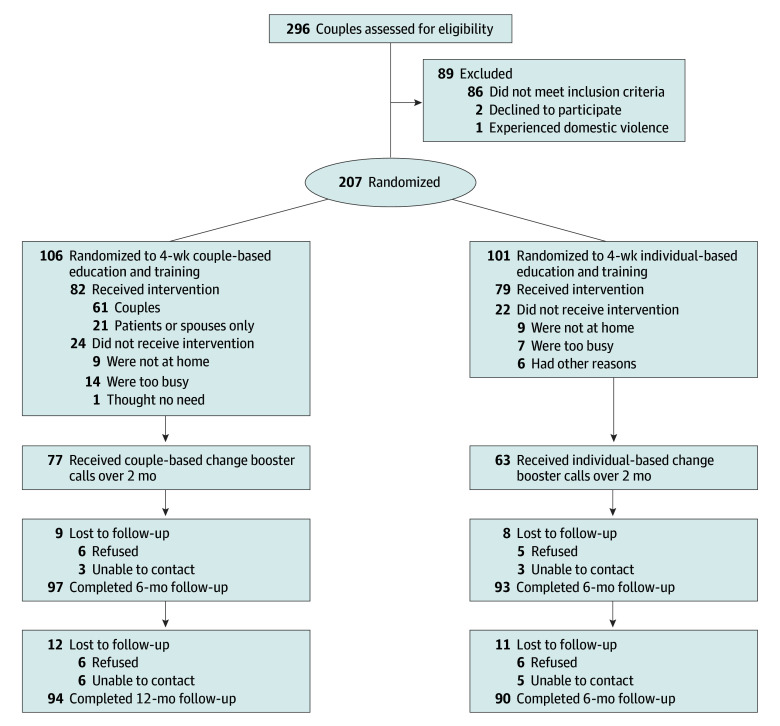
Participant Flow Through the Intervention

Most spouses in both arms appraised diabetes management as a shared responsibility (intervention arm, 65 [61.3%]; control arm, 55 [54.5%]) (eTable 2 in Supplement 2). Both arms had similar follow-up rates at 6 and 12 months (intervention arm, 91.5% and 88.7%, respectively; control arm, 92.1% and 89.1%, respectively) (Figure 1).

### Treatment Effect Across Outcomes Over Follow-Up

Patients’ HbA_1c_ levels in both arms decreased over the 12-month follow-up (eTable 1 in Supplement 2). However, changes did not reach statistical significance either within each arm or between arms (β = −0.01 [95% CI, −0.52 to 0.50] at 6 months and β = −0.08 [95% CI, −0.57 to 0.42] at 12 months). No statistically significant differences were found in quality of life for patients and their spouses. Both the PCS and MCS scores of the SF-36 remained stable over time (Table 2).

**Table 2.** Health Outcomes of Patients and Spouses at Baseline and Follow-Up Between the Intervention and Control Arms

Outcome	β (95% CI)		
	Intervention arm	Control arm	Difference between arms^a^
**Patients**			
HbA_1c_, %			
Baseline	8.61 (8.15 to 9.07)	8.77 (6.62 to 10.92)	−0.16 (−0.62 to 0.30)
Follow-up after 6 mo	8.62 (7.74 to 9.49)	8.62 (8.26 to 8.99)	−0.01 (−0.52 to 0.50)
Follow-up after 12 mo	8.59 (7.73 to 9.45)	8.67 (8.30 to 9.03)	−0.08 (−0.57 to 0.42)
SF-36 PCS score			
Baseline	71.01 (67.81 to 74.21)	69.94 (53.27 to 86.61)	1.07 (−2.13 to 4.27)
Follow-up after 6 mo	68.99 (61.35 to 76.63)	70.37 (67.14 to 73.60)	−1.38 (−5.80 to 3.03)
Follow-up after 12 mo	70.39 (62.76 to 78.02)	70.48 (67.14 to 73.81)	−0.09 (−4.38 to 4.21)
SF-36 MCS score			
Baseline	72.60 (70.13 to 75.08)	71.28 (61.21 to 81.35)	1.32 (−1.15 to 3.80)
Follow-up after 6 mo	72.14 (66.57 to 77.71)	73.43 (71.11 to 75.75)	−1.29 (−4.54. 1.95)
Follow-up after 12 mo	71.11 (65.55 to 76.66)	73.52 (71.17 to 75.87)	−2.41 (−5.62 to 0.79)
**Spouses**			
SF-36 PCS score^b^			
Baseline	63.27 (60.53 to 66.02)	63.40 (50.93 to 75.86)	−0.12 (−2.87 to 2.62)
Follow-up after 12 mo	63.37 (56.26 to 70.47)	61.73 (58.70 to 64.77)	1.63 (−2.44 to 5.71)
SF-36 MCS score^b^			
Baseline	71.46 (69.26 to 73.67)	71.85 (62.35 to 81.35)	−0.39 (−2.60 to 1.82)
Follow-up after 12 mo	71.60 (66.04 to 77.16)	72.22 (69.86 to 74.58)	−0.62 (−3.82 to 2.58)

Abbreviations: HbA_1c_, hemoglobin A_1c_; MCS, mental component summary; PCS, physical component summary; SF-36, 36-Item Short Form Health Survey.

^a^ Results were obtained by fitting a multilevel model with the control arm as the reference. Age, sex, education, diabetes duration, and retirement were adjusted in patients; age, sex, education, and retirement were adjusted in spouses.

^b^ Responses for the SF-36 were not collected from spouses at the 6-month follow-up.

Statistically significant changes were found in collective efficacy and behavior among patients such that C-DMSES scores, SDSCA scores, and physical activity levels in terms of metabolic equivalent tasks all increased over follow-up compared with baseline for both the intervention and control arms (Table 3). However, the magnitude of these changes was similar between arms, rendering no between-arm differences. Couples’ type 2 diabetes management patterns, as assessed by an illness appraisal questionnaire completed at baseline and 12-month follow-up, also remained stable (eTable 2 in Supplement 2). None of these measures differed among spouses of either arm.

**Table 3.** Collective Efficacy and Behaviors of Patients and Spouses at Baseline and Follow-Up Between the Intervention and Control Arms

Variable	β (95% CI)					
	Patients^a^			Spouses^b^		
	Intervention arm	Control arm	Difference between arms	Intervention arm	Control arm	Difference between arms
**Collective efficacy**						
C-DMSES						
Baseline	141.0 (131.3 to 150.8)	143.3 (98.6 to 187.9)	−2.2 (−12.0 to 7.5)	114.7 (100.2 to 129.3)	115.6 (47.9 to 183.2)	−0.8 (−15.4 to 13.7)
Follow-up after 6 mo	160.9 (140.6 to 181.2)^c^	158.7 (150.2 to 167.3)^c^	2.1 (−9.6 to 13.9)	NA	NA	NA
Follow-up after 12 mo	159.9 (139.4 to 180.4)^c^	162.3 (153.6 to 170.9)^c^	−2.4 (−14.3 to 9.5)	134.4 (101.9 to 166.9)	122.9 (109.2 to 136.7)	11.5 (−7.3 to 30.2)
**Collective behaviors**						
SDSCA						
Baseline	22.5 (18.7 to 26.3)	23.6 (8.0 to 39.1)	−1.1 (−4.9 to 2.7)	NA	NA	NA
Follow-up after 6 mo	29.4 (20.9 to 37.9)^c^	29.1 (25.5 to 32.6)^c^	0.4 (−4.5 to 5.3)	NA	NA	NA
Follow-up after 12 mo	28.6 (19.7 to 37.5)	27.2 (23.3 to 30.9)	1.5 (−3.7 to 6.6)	NA	NA	NA
**Physical activity, MET-min/wk**						
Baseline	3398.1 (2848.2 to 3948.1)	3367.4 (1113.8 to 5621.0)	30.8 (−519.2 to 580.7)	2743.6 (2036.9 to 3450.2)	2244.6 (−770.9 to 5260.1)	499.0 (−207.7 to 1205.6)
Follow-up after 6 mo	4008.4 (2779.2 to 5237.5)^c^	3930.7 (3421.2 to 4440.2)^c^	77.7 (−642.0 to 797.3)	NA	NA	NA
Follow-up after 12 mo	3567.8 (2336.4 to 4799.2)	3866.1 (3352.3 to 4379.9)	−298.3 (−1015.9 to 419.3)	2046.9 (217.3 to 3876.4)	2168.0 (1378.5 to 2957.5)	−121.1 (−1161.2 to 919.0)

Abbreviations: C-DMSES, Chinese version of the Diabetes Management Self-Efficacy Scale; MET, metabolic equivalent task; NA, not applicable; SDSCA, Summary of Diabetes Self-Care Activities.

^a^ Results were obtained by fitting a multilevel model, which adjusted for age, sex, education, diabetes duration, and retirement, with the control arm as the reference. Within arms, baseline was used as control.

^b^ Results were obtained by fitting a multilevel model, which adjusted for age, sex, education, and retirement, with the control arm as the reference. Responses for C-DMSES, SDSCA, and physical activity were not collected from spouses at the 6-month follow-up.

^c^ Significant difference.

### Subgroup Analyses by Sex, Baseline HbA_1c_ Level, and Intervention Fidelity

**Figure 2.**
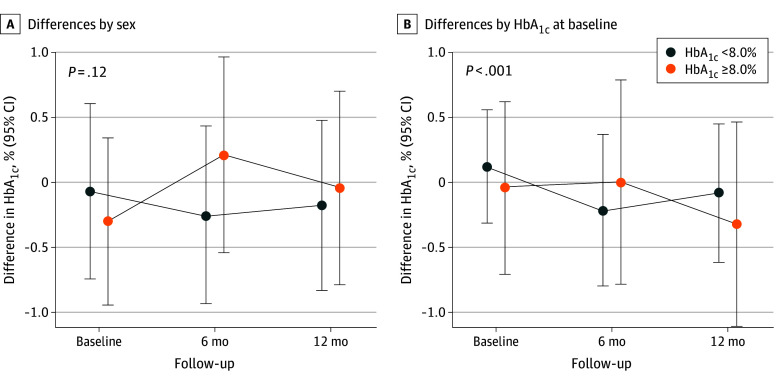
Subgroup Differences in Patients’ Hemoglobin A_1c_ (HbA_1c_) Between Intervention and Control Arms From Baseline to Follow-Up The difference in HbA_1c_ was calculated as the intervention arm minus the control arm. The *P* values for differences in change trend are by preset subgroup analyses.

Despite divergent HbA_1c_ trends between arms for men and women across repeated measures, there was no statistically significant difference by sex (*P* for interaction = .12). However, decreases in HbA_1c_ between arms were more constant and lasting in patients with a high baseline HbA_1c_ levels (ie, ≥8.0%) in contrast to a reverse trend observed for patients with HbA_1c_ less than 8.0% at baseline, leading to a significant difference by baseline HbA_1c_ (*P* < .001 for interaction) (Figure 2).

## Discussion

Guided by a theoretical framework, our RCT was designed to enhance the collective efficacy and collective behavior of couples to improve glycemic control in patients with type 2 diabetes and the couples’ overall quality of life. Partially consistent with our hypothesis, we identified improvements in collective efficacy and behaviors among patients in both arms compared with baseline. However, we did not find statistically significant differences in either HbA_1c_ or quality of life between the couple-based intervention and individual-based control arm. A statistically significant between-arm difference in HbA_1c_ reduction was found in the subgroup analysis by patients’ baseline HbA_1c_ level.

We failed to identify any statistically significant between-arm differences in health outcomes. The insignificant difference in HbA_1c_ may have resulted from greater heterogeneity among the enrolled patients than anticipated in combination with a smaller mean difference between arms. Based on these parameters, a sample size at least 3 times larger is needed to detect the treatment effect. Nevertheless, nonstatistically significant between-arm differences in HbA_1c_ have been commonly seen in couple-based RCTs. Prior studies by Trief et al^11^ and Wing et al^12^ reported no significant changes in either HbA_1c_ or fasting glucose levels. Of the 25 studies systematically reviewed by Pratiwi et al,^31^ only 1 reported a significant between-arm difference in HbA_1c_ levels. These investigators, however, conducted a family-based education intervention led by pharmacists, with the control group receiving usual care, rendering a mixed effect of health education and spousal collaboration.^32^ Regarding quality of life, both arms showed stability in SF-36 scores, which may be due to our study’s short duration. This finding is consistent with the findings of Miklavcic et al,^33^ who reported no significant change in PCS and MCS scores in the short term after intervention among older adults with a long duration of type 2 diabetes and multiple comorbidities.

Taken together, findings from our study and prior RCTs indicate that the treatment effect of couple-based intervention is weak. Possible reasons for this weak or lack of treatment effect may include the nature of couple-based intervention (ie, the treatment effect is purely added value of spousal involvement in diabetes management).^11^ This effect, if there is any, would be less evident than that of structured diabetes self-management education.^34^ Given that both arms received group education, the extra effect attributable to the spouse may be difficult to detect with a limited sample size.^11^ Particularly against the Chinese background, whereby family harmony and cohesion are highly valued, spousal involvement in patients’ daily care is prevalent.^35^ In our study sample, 61.3% and 54.5% of spouses in the intervention and control arms, respectively, appraised diabetes management as a responsibility in which they should take part. This preexisting high involvement of spouses may further dilute the expected effect of couple-based intervention.

Moreover, the treatment effect of couple-based intervention largely depends on the degree to which couples modify their management patterns and behaviors as required. Despite within-arm changes in terms of patients’ collective efficacy and behavior, no differences were found between arms. Couples’ diabetes management patterns also stayed stable. These findings suggest that the difficulty of reversing long-standing habits established among older couples may be a challenge for the couple-based intervention.^36^ As a previous study by Zheng et al^37^ found, when the patient and spouse both appraised diabetes as a shared problem, dyadic coping was perceived more often and was associated with diabetes management efficacy. It is possible that the intervention may help increase understanding and perceived support from spouses by involving them in the education and behavior change process, but it may not translate into actual support to actively or sufficiently alter their habits.^38^ Interviews conducted by Zhang et al^39^ with the study couples further revealed that the successful implementation of a couple-based intervention is conditional on the couple’s preference. The extent that the attendance rate of patients in the intervention arm was lower than in the control arm demonstrated particularly low spousal support, a finding that will be reported and discussed in a future publication. Couples’ joint attendance and behavior change were also affected by the lockdown due to COVID-19.^40^

Although the overall effect of couple-based intervention is weak, this intervention may be beneficial for older patients with difficult-to-control glycemic levels. In line with the findings of Trief et al,^11^ our study showed that the couple-based intervention yielded more pronounced and longer-lasting glycemic control among patients with an HbA_1c_ level greater than 8.0% than those with better control (HbA_1c_ <8.0%) at baseline. Previous studies have shown that the challenges faced by older adults with poorly controlled HbA_1c_, including low adherence to treatment regimens, may lead to high risks of cardiovascular diseases, geriatric syndromes, and premature death if unaddressed.^41,42^ These individuals often require additional support to achieve glycemic targets, underscoring the importance of mobilizing their families for diabetes management, as exemplified by couple-based intervention.^11^

### Strengths and Limitations

Our multicentered RCT with adequate response rates contributes to the evidence on couple-based intervention in an East Asian older population with type 2 diabetes. The intervention was theoretically grounded and evaluated the added value of spousal involvement in diabetes management from both patients’ and spouses’ perspectives repeatedly assessed throughout the 12-month study period.

Nonetheless, the study has several limitations. First, our study participants may have represented couples with relatively good relationships. Older couples with low interest in collaborating with each other may find it difficult to be involved in this kind of intervention.^43^ Second, our inclusion criterion for HbA_1c_ level was defined broadly to maximize the number of patients to be included. This approach, however, resulted in great heterogeneity in participants’ HbA_1c_ levels compared with previous studies. Although we used a multilevel model to control for patient- and community-level heterogeneity as random effects, plus additional adjustment for baseline characteristics, it was still difficult to find the expected between-arm differences given the large variation with the existing sample size. Finally, collective efficacy and behavior were measured by self-report, which may have incurred reporting bias.

## Conclusions

Our multicenter RCT in a clinical setting evaluated the effect of a couple-based intervention among community-dwelling Chinese older adults with type 2 diabetes and their spouses. The detected treatment effect was weak in general but was found to be beneficial for older adults with poorly controlled glycemia. Future research and practice should consider tailored diabetes management strategies that match the patients’ characteristics while taking their spouses’ availability and preference for support into consideration.

## Notes

Supplement 1.
Trial Protocol

Supplement 2. **eFigure.** Theoretical Framework for Couples-Based Intervention **eTable 1.** Model Comparison **eTable 2.** Intervention Implementation Characteristics

Supplement 3.
Data Sharing Statement
