# Supplementary material for: A Couple-Based Intervention for Chinese Older Adults With Type 2 Diabetes: A Randomized Clinical Trial
Source: JAMA Netw Open. 2025 Jan 2;8(1):e2452168. doi: 10.1001/jamanetworkopen.2024.52168 (PMC11696449; doi:10.1001/jamanetworkopen.2024.52168)
Supplement: Supplement 1. — Trial Protocol [file jamanetwopen-e2452168-s001.pdf]

School of Public Health, Sun Yat-sen University

Ethical Review of Medical Research Application Form

Project Title: Development and Validation of Couple Collaborative Management Model of Chronic Diseases for Community-Dwelling Older Adults

Project Category: National Natural Science Foundation

Project Duration: January 2019 to December 2021

Applicant: Jing Liao

Contact Number: 020-84112657

Email: liaojing5@mail.sysu.edu.cn

Date: March 6, 2019

Issued by the Medical Ethics Committee of the School of Public Health, Sun Yat-sen University

Research Purpose and Significance

Research Purpose:

Taking type 2 diabetes as an example, this project aims to construct a collaborative management model for chronic diseases among elderly couples in the community and to verify the effectiveness of this model when applied to elderly patients with chronic diseases living at home in China.

Research Significance:

Against the backdrop of the increasing number of empty-nest elderly households in China, a major challenge faced by healthcare professionals is to develop an effective management model for chronic diseases among the elderly that integrates the physiological and psychological characteristics of elderly Chinese individuals, thereby improving their quality of life. This study seeks to establish a collaborative management model for chronic diseases among elderly couples in the community, tailored to the conditions in China. This model aims to provide new solutions for the issue of chronic disease patients knowing what to do but not acting on it, or not sustaining their actions. It will also offer new ideas for primary prevention among high-risk elderly populations and for reducing caregiving burdens.

The study will offer empirical evidence on how to maximize the use of limited community health resources and efficiently conduct chronic disease prevention and treatment among elderly community residents. Furthermore, the findings of this research will be valuable for China's ongoing implementation of the family doctor system. By focusing on the primary target population of family doctor contracts—elderly community residents—the study will provide a comprehensive understanding of the health status, self-management challenges, and needs of elderly chronic disease patients and their spouses. This will offer firsthand evidence for family doctors to further fulfill their roles at the family level.

Research Funding

The required funding for the research is 195,000 RMB, which is supported by the National Natural Science Foundation of China.

Profiles of Principal and Participating Researchers

number	name	degree	position	divixion	affiliation
1	Xiongfei Chen	Master	Chief Physician	Organization and coordination of on-site work, supervision	Guangzhou Center for Disease Control and Prevention
2	Xueji Wu	Master	Deputy Chief Physician		
3	Shaolong Wu	Doctor	Associate Professor	Qualitative interviews, expert consultation in the health field	Sun Yat-sen University
4	Yongren Yang	Master	Attending Physician	Guidance on elderly medicine-related content	Taiwan Society of Geriatric Psychiatry
5	Li Cai	Doctor	Lecturer	Guidance on nutrition-related issues	Sun Yat-sen University

Research Methods

Study Design and Procedures:
The study will employ a cluster-randomized controlled trial design to evaluate the effectiveness and economic benefits of a collaborative management model for elderly diabetes patients living at home in China. The study will be conducted across 20 community health centers in Guangzhou, recruiting 194 pairs of elderly diabetes patients (aged 55 and above) and their spouses from these centers.

Participants will be randomly allocated 1:1 within each community health center to either the collaborative intervention group (couples) or the control group (patients only).

Baseline Assessment:
At baseline, both the intervention and control groups will undergo physical examinations and complete relevant questionnaires. During the study, diabetes patients will continue their standard treatment regimen.

Intervention:
Intervention Group: Couples will participate in a 4-week health education program (weekly, 1.5 hours per session) followed by a 2-week behavioral skills training.
Control Group: Only the patients will receive the same intervention components as those in the intervention group.
Both groups will follow identical management modules, intervention durations, and frequencies. The key distinction is that the intervention group will receive the

intervention as a couple, while the control group will receive it individually.

Follow-Up:
Follow-ups will be conducted at 3-, 6-, and 12-months post-intervention.

Outcome Measures:
Patients: Hemoglobin A1c levels
Spouses: Quality of life
Process Measures:
Data will also be collected on the collaborative coping and management effectiveness, basic demographic information, health status, diabetes knowledge levels, and support behaviors for both patients and their spouses.

Statistical Methods:
Analysis: Intention-to-treat analysis will be employed. Differences between the intervention and control groups will be assessed using multilevel linear models.
Theoretical Model: Structural equation modeling will be used to explore the applicability of the theoretical model.
Cost-Benefit Evaluation: The intervention results will be subject to a cost-benefit analysis to evaluate economic implications.

This comprehensive approach aims to rigorously assess the impact of the collaborative management model on both the effectiveness and economic efficiency in managing chronic diseases among elderly couples in a community setting.

Participants

Total Number of Participants:
The study will involve 194 pairs of elderly diabetes patients (aged 55 and above) and their spouses, who are receiving care at 20 community health centers and participating in elderly health management and diabetes management programs.

For Patients:
Inclusion Criteria: ①Diagnosis of type 2 diabetes according to WHO criteria (1999), with a confirmed diagnosis; ②Aged ⩾55 years; ③Possess full cognitive and behavioral abilities; ④Reside with their spouse; ⑤Provide informed consent and volunteer to participate in the study. Exclusion Criteria: ①Acute complications of diabetes; ②Unclear consciousness, uncooperative behavior, or impaired language expression；③Severe concomitant conditions such as major heart, brain, or lung diseases；④Physical activity limitations due to complications or comorbidities.

For Spouses:
①Reside with the elderly diabetes patient as their spouse/partner；②No severe physical illnesses or mental disorders；③Capable of independent living；④Provide informed consent and volunteer agree to participate in the study.

If both partners are diabetes patients, the qualified research subject will be selected based on the community chronic disease management system, prioritizing those identified as suitable for inclusion.

Potential Adverse Reactions or Events and Mitigation Strategies

Adverse Reactions:
This study is conducted in accordance with health management protocols and aligns with the latest clinical guidelines for diabetes. It is overseen by qualified clinical professionals and is not expected to cause adverse reactions. However, if participants experience any personal health issues that prevent them from continuing in the study, they may withdraw after informing the research team.

Potential Adverse Events and Management Strategies:
Schedule Conflicts and Privacy Concerns:
Issue: There may be conflicts between the intervention schedule and participants' personal schedules. Additionally, the topics discussed during surveys and interventions may involve sensitive issues that could make participants uncomfortable.
Mitigation: All healthcare providers involved in delivering health education and behavioral interventions will undergo standardized training to ensure respectful communication. Participants will be asked for consent before discussing sensitive or private topics. They can opt out of discussing certain topics or discontinue participation in interventions at any time without facing any penalties. All survey data will be anonymized to protect participant privacy. Research results will be used solely for scientific purposes. Personal health information and treatment results will be kept confidential, with no disclosure to unauthorized individuals without participant consent.
Blood Sample Collection:
Issue: The collection of blood samples by community general practitioners may cause minor skin damage and carry a risk of infection.
Mitigation: Blood sample collection will be performed by trained community general practitioners using sterile tools to minimize infection risk. The volume of blood collected will be minimal to reduce physical harm to participants.

These strategies aim to ensure participant safety and confidentiality while minimizing any potential risks associated with the study.

Statement of Adherence to Ethical Principles

We hereby affirm our commitment to adhere to the ethical principles outlined in relevant guidelines for this research study. This includes compliance with ethical guidelines, acceptance of ethical committee requirements, submission of progress reports, notification of protocol and consent form modifications, participant informed consent, etc

Signature of the Principal Investigator：

March 6, 2019

Opinion of the Ethics Committee of the School of Public Health:

Seal of Director:

(Month, Day, Year)
