# Supplementary material for: A Couple-Based Intervention for Chinese Older Adults With Type 2 Diabetes: A Randomized Clinical Trial
Source: JAMA Netw Open. 2025 Jan 2;8(1):e2452168. doi: 10.1001/jamanetworkopen.2024.52168 (PMC11696449; doi:10.1001/jamanetworkopen.2024.52168)
Supplement: Supplement 2. — eFigure. Theoretical Framework for Couples-Based Intervention eTable 1. Model Comparison eTable 2. Intervention Implementation Characteristics [file jamanetwopen-e2452168-s002.pdf]

Supplemental Online Content

Yang C, Zhi J, Xu Y, et al. A couple-based intervention for Chinese older adults with type 2 diabetes: a randomized clinical trial. *JAMA Netw Open*. 2025;8(1):e2452168. doi:10.1001/jamanetworkopen.2024.52168

eFigure. Theoretical Framework for Couples-Based Intervention

eTable 1. Model Comparison

eTable 2. Intervention Implementation Characteristics

This supplemental material has been provided by the authors to give readers additional information about their work.

© 2025 Yang C et al. *JAMA Network Open*.

eFigure. Theoretical Framework for Couples-Based Intervention (+ Enhanced, − Reduced)

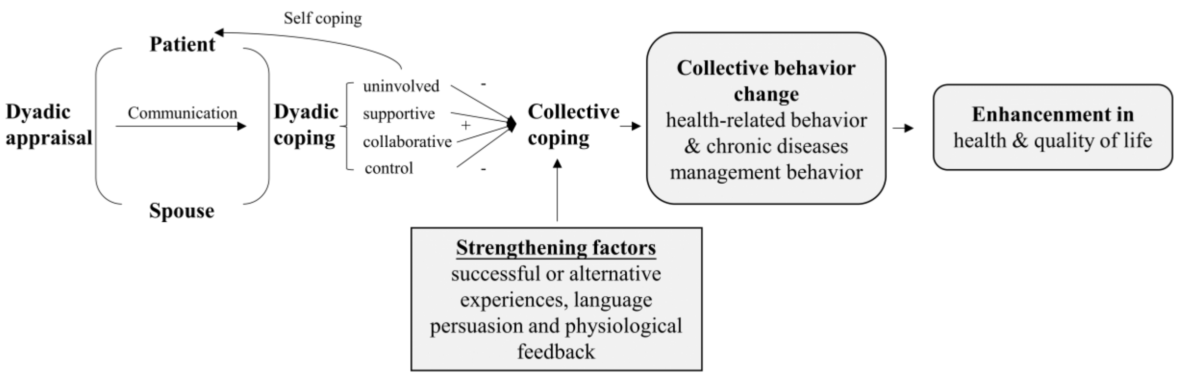

The figure cited from Liao, J., Wu, X., Wang, C. *et al.* Couple-based collaborative management model of type 2 diabetes mellitus for community-dwelling older adults in China: protocol for a hybrid type 1 randomized controlled trial. *BMC Geriatrics* **20**, 123 (2020). https://doi.org/10.1186/s12877-020-01528-5

© 2025 Yang C et al. *JAMA Network Open*.

eTable 1. Model Comparison

hemoglobin A1C (HbA1C)（%）	Model 0<br>$\beta(SE)$	Model 1<br>$\beta(SE)$	Model 2<br>$\beta(SE)$
Intercept	8.46 (0.19)	8.78 (1.10)	8.79 (1.14)
Intervention arm	-0.25 (0.24)	-0.16 (0.23)	-0.24 (0.17)
Wave			
Follow-up after 6 months	-0.26 (0.17)	-0.24 (0.17)	-0.11 (0.16)
Follow-up after 6 months	-0.12 (0.16)	-0.11 (0.16)	-0.16 (0.23)
Age	-	-0.02 (0.02)	-0.02 (0.02)
Male	-	0.14 (0.21)	0.14 (0.21)
Education [1]			
Secondary school	-	-0.28 (0.26)	-0.24 (0.26)
High school and above	-	-0.4 (0.26)	-0.36 (0.26)
Retired	-	0.69 (0.32)	0.68 (0.32)
Diabetes duration	-	0.07 (0.01)	0.07 (0.01)
Attendance number [2]			
Telephone completion rate [3]			
Intervention arm * Follow-up after 6 months	-0.02 (0.23)	-0.04 (0.23)	-0.04 (0.23)
Intervention arm * Follow-up after 12 months	-0.06 (0.23)	-0.09 (0.23)	-0.08 (0.23)
Goodness-of-fit test			
AIC	2426.23	2400.24	2410.65
BIC	2466.12	2462.28	2469.39
Likelihood ratio test	Reference	<0.001	<0.001

[1] Reference at primary school and below
"-", Not applicable.

© 2025 Yang C et al. *JAMA Network Open*.

eTable 2. Intervention Implementation Characteristics

	Time Since Baseline	Operationalization		Patients			Spouses		
				Intervention Arm (N=106)	Control Arm (N=101)	*P*	Intervention Arm (N=106)	Control Arm (N=101)	*P*
Fidelity	Over 1 month	Checklist evaluating the Implementation of education, ranging 0 to 1, mean (SD)		0.9 (0.1)	0.9 (0.1)	0.74	-	-	-
Attendance number	Over 1 month	Number of education sessions attendance, with 4 sessions in total		2.1	2.7	0.03	1.9	-	
Telephone completion rate	Over 2-3 months	Response rate during behavior change booster		82.1	86.1	0.69	65.1	-	-
Satisfaction	After 3 months	Participants' satisfaction with the intervention, ranging 1 to 6, mean (SD)		5.5 (0.3)	5.4 (0.5)	0.51	-	-	-
Follow up rate	After 6 months	Response rate during follow up		91.5	92.1	1	85.9	-	-
	After 12 months			88.7	89.1	1	74.5	64.4	0.09
Illness appraisal	At baseline	Illness appraisals questionnaire [a]	Patient issue only	49.1	45.5	0.92	23.6	26.7	0.51
			Patient issue but affects the spouse	27.1	25.7		15.1	17.8	
			Shared issue	28.3	27.7		61.3	54.5	
			Spouse issue only	0.9	1.0		0.0	1.0	
	After 12 months		Patient issue only	39.6	32.1	0.26	19.5	21.4	0.71
			Patient issue but affects the spouse	29.7	22.6		12.2	7.1	
			Shared issue	29.7	44.0		65.9	70.0	
			Spouse issue only	1.1	1.2		2.4	1.4	

[a] Zheng, H., Liu, Y., Cai, Y.. et al. The reliability and validity of the couple collaborative management scale for middle-aged and older adults with type 2 diabetes mellitus. *Chin J Health Educ* 39(01): 41–46 (2023). https://doi.org/10.16168/j.cnki.issn.1002-9982.2023.01.008

© 2025 Yang C et al. *JAMA Network Open*.
