# Supplementary material for: A Couple-Based Intervention for Chinese Older Adults With Type 2 Diabetes: A Randomized Clinical Trial
Source: JAMA Netw Open. 2025 Jan 2;8(1):e2452168. doi: 10.1001/jamanetworkopen.2024.52168 (PMC11696449; doi:10.1001/jamanetworkopen.2024.52168)
Supplement: Supplement 3. — Data Sharing Statement [file jamanetwopen-e2452168-s003.pdf]

Data Sharing Statement

Yang. A Couple-Based Intervention for Chinese Older Adults With Type 2 Diabetes. *JAMA Netw Open*. Published January 02, 2025. doi:10.1001/jamanetworkopen.2024.52168

Data
Additional Information: Trial Registration: Chinese Clinical Trial Registry (https://www.chictr.org.cn/indexEN.html), RCT no. ChiCTR1900027137, Registered 1st Nov. 2019.
Data available: Yes
Data types: Deidentified participant data
How to access data: Public access to the study raw data will be available upon approval via the Chinese Clinical Trial Registry(https://www.chictr.org.cn/aboutEN.html) or from the corresponding author(liaojing5@mail.sysu.edu.cn) upon reasonable request.
When available: With publication

Supporting Documents
Document types: None

Additional Information
Who can access the data: Researchers whose proposed use of the data has been approved.
Types of analyses: For any purpose.
Mechanisms of data availability: After approval of a proposal or with a signed data access agreement and with investigator support.
